# Ion Channel Dysfunction and Neuroinflammation in Migraine and Depression

**DOI:** 10.3389/fphar.2021.777607

**Published:** 2021-11-10

**Authors:** Emine Eren-Koçak, Turgay Dalkara

**Affiliations:** ^1^ Institute of Neurological Sciences and Psychiatry, Hacettepe University, Ankara, Turkey; ^2^ Department of Psychiatry, Medical Faculty, Hacettepe University, Ankara, Turkey

**Keywords:** ATP1A2, HCN, KCNQ, CACNA, TREK, Pannnexin-1, P2X7R, neuroinflammation

## Abstract

Migraine and major depression are debilitating disorders with high lifetime prevalence rates. Interestingly these disorders are highly comorbid and show significant heritability, suggesting shared pathophysiological mechanisms. Non-homeostatic function of ion channels and neuroinflammation may be common mechanisms underlying both disorders: The excitation-inhibition balance of microcircuits and their modulation by monoaminergic systems, which depend on the expression and function of membrane located K^+^, Na^+^, and Ca^+2^ channels, have been reported to be disturbed in both depression and migraine. Ion channels and energy supply to synapses not only change excitability of neurons but can also mediate the induction and maintenance of inflammatory signaling implicated in the pathophysiology of both disorders. In this respect, Pannexin-1 and P2X7 large-pore ion channel receptors can induce inflammasome formation that triggers release of pro-inflammatory mediators from the cell. Here, the role of ion channels involved in the regulation of excitation-inhibition balance, synaptic energy homeostasis as well as inflammatory signaling in migraine and depression will be reviewed.

## Introduction

Migraine and depression are comorbid diseases (Amiri et al., 2019; Karsan and Goadsby, 2021). Migraine is a risk factor for depression, whereas migraine attack prevalence and intensity increase during depression. Despite this well-established epidemiological data, the biological basis of this comorbidity is unclear. Emerging data from animal models and human imaging studies suggest that neuroinflammation could be a common pathway in the pathophysiology of both disorders (Mueller and Schwarz, 2007; Richards et al., 2018; Albrecht et al., 2019; Hadjikhani et al., 2020; Afridi and Suk, 2021). However, how neuroinflammatory signaling is initiated without an injury to the brain tissue is unknown. A non-homeostatic synaptic transmission has been suggested as an initiator of neuroinflammatory signaling (Dalkara and Kilic, 2013; Kilic et al., 2018; Afridi and Suk, 2021; Petit et al., 2021). In this regard, some ion channels and activation of cellular stress sensors such as pannexin1 (Panx1) and P2X7 channels and the downstream inflammatory cascade may play a role. Here, we review the recent evidence suggesting that a non-homeostatic ion channel activity or a mismatch between synaptic energy supply and glutamatergic transmission can activate the inflammatory pathway in migraine and depression. Since we limit the scope of the review to these potentially common central mechanisms in both disorders, we will not be able to cover recent developments regarding the success of CGRP antagonists as novel anti-migraine drugs and their implications for peripheral nociceptive mechanisms of migraine headache.

### Lessons Learned From Familial Migraine Mutations

**FIGURE 1:**
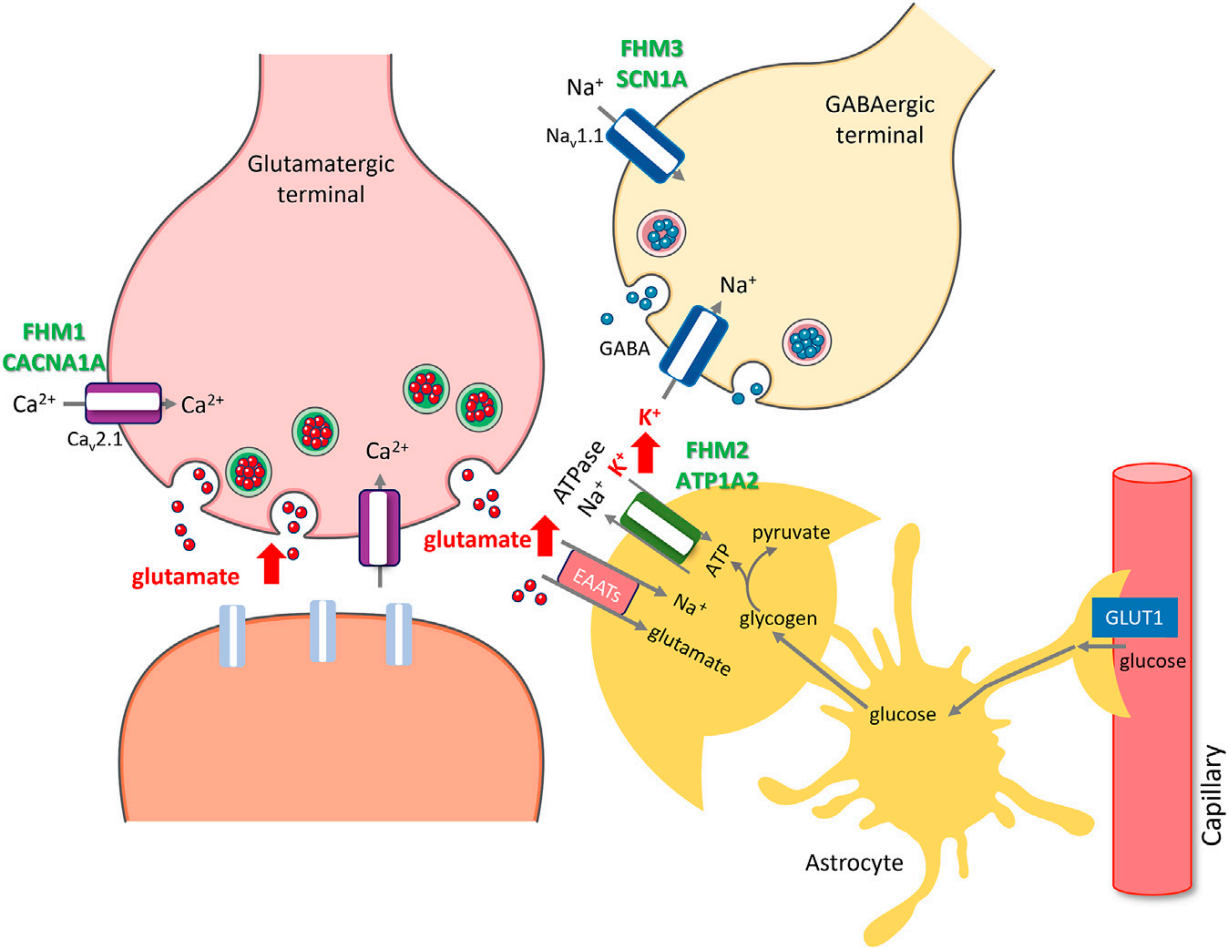
Rises in extracellular glutamate and K+ caused by ion channel and transporter mutations or transcriptional changes induced by migraine triggers create susceptibility to migraine with aura. Facilitation of excitatory synaptic transmission by either promoting glutamate release (red arrows) due to gain of function mutations in P/Q type presynaptic calcium channels (purple) encoded by CACNA1A in FHM1 or decreasing its uptake along with K+ (red arrow) due to mutations in alpha2 subunit of Na+/K + ATPase (green) encoded by ATP1A2 in FHM2, increases susceptibility to CSD and migraine with aura. Glutamate released during synaptic activity is taken up to astrocyte processes (amber) by excitatory amino acid transporters (pink, EAATs), which is driven by the Na + gradient created by Na+/K + ATPase, hence, glutamate and K+ uptakes are coupled. Excess K+ released from overactive GABAergic terminals (red arrow) due to gain of function mutations in Na_V_ 1.1 type sodium channels (blue) encoded by SCN1A in FHM3 also creates susceptibility to CSD and migraine with aura. Glycogen granules in peri-synaptic astrocyte processes instantly provide glycosyl units to meet the rapidly escalating energy demand during excitatory synaptic transmission (e.g., by Na+/K + ATPase), whereas glucose transported from circulation to astrocytes via GLUT1 is primarily used in replenishing glycogen. Insufficient glycogen breakdown due to transcriptional changes induced by migraine triggers such as sleep deprivation, which promote glycogen synthesis while reducing glycogen breakdown, hinders glutamate and K+ uptake as seen in FHM2, creating susceptibility to CSD and migraine in non-familial common migraine. The illustration is prepared by Dr. Zeynep Kaya. Cellular templates are copied from Servier Medical Art (smart.servier.com).

Most of our knowledge about the potential role of ion channels and glutamate in migraine comes from the discovery of mutant genes causing familial hemiplegic migraine (FHM) (Moskowitz et al., 2004; Russell and Ducros, 2011; Pietrobon and Moskowitz, 2014; Dalkara and Moskowitz, 2017; De Boer et al., 2019; Pietrobon and Brennan, 2019). FHM patients suffer from typical migraine with aura episodes along with transient hemiplegic or hemiparetic attacks (Russell and Ducros, 2011). Some patients may also experience confusion, memory loss, seizures and coma episodes and, rarely, persistent deficits like ataxia. However, these severe phenotypes are typical of FHM1 and 2 but not of FHM3 (Mantegazza, 2018) and, other than episodic attacks, majority of FHM 1 and 2 patients are healthy except being more sensitive to head trauma. Hemiplegic attacks and an autosomal dominant inheritance pattern distinguish them from non-familial, common forms of migraine (Sutherland H. G. et al., 2019). Although they are rare and the mutations have yet been detected in less than half of the cases (Sutherland et al., 2020), the three genes identified so far have provided insight into how a migraine attack can be initiated in an otherwise healthy brain (Moskowitz et al., 2004; Pietrobon and Brennan, 2019). Mutations in *CACNA1A, ATP1A2,* and *SCN1A* genes, which encode a P/Q-type voltage-gated calcium channel (Ca_V_2.1), an astrocytic Na/K pump (α2 Na/K-ATPase) and the α1 subunit of voltage-gated Na_V_1.1 sodium channel, account for FHM1, FHM2 and FHM3, respectively (Figure 1). Of these, *CACNA1A*, *ATP1A2* mutations have been comprehensively studied with regard to their effects on cortical excitability (for review, (Pietrobon and Brennan, 2019). *In vitro* and *in vivo* evidence suggests that *CACNA1A* mutations can lead to enhanced glutamate release during excitatory synaptic activity because gain-of-function mutations on the pore-forming α1-subunit of the presynaptic P/Q type calcium channels enable them to open at more hyperpolarized membrane potentials than normal, allowing more calcium influx to the terminal during action potential trains (Tottene et al., 2009). Interestingly, the P/Q type calcium channels on terminals of GABAergic neurons are not affected by these mutations (Vecchia et al., 2014). This causes a shift in cortical excitation/inhibition balance towards excitation (Vecchia and Pietrobon, 2012; Tottene et al., 2019), which is thought to account for the vulnerability to cortical spreading depolarization (CSD), the putative cause of migraine aura and, for the hyperexcitability seen in FHM1 brain (Takizawa et al., 2020a). Given the presence of glutamatergic synapses driving the feed-forward and feed-back inhibitory neurons, one may expect an increased inhibitory control as well. However, this is probably not the case owing to the fact that short-term depression develops faster in these glutamatergic synapses than does in glutamatergic synapses terminating on principal neurons (Tottene et al., 2019). Although the net effect of the excitation/inhibition balance might be different in various neuronal circuits depending on the organization of neuronal interconnections and release kinetics of synapses, including pain-transmitting/processing networks, it has been clearly shown that the CSD threshold is decreased in transgenic knock-in mice harboring human *CACNA1A* mutations (Van Den Maagdenberg et al., 2004). In fact, the threshold was found to be even lower in *S218L* compared to *R192Q* knock-in mouse in parallel with the clinical severity of FHM symptoms in patients carrying these mutations (Van Den Maagdenberg et al., 2010). However, some FHM1 and FHM2 patients also suffer from epileptic seizures suggesting that excitation/inhibition imbalance has also potential to generate epileptic discharges (Marconi et al., 2003; Lebas et al., 2008; Costa et al., 2014).

Cortical spreading depolarization is considered the electrophysiological correlate of migraine aura (Takizawa et al., 2020a). First discovered by Leao in 1944 on the rabbit brain, it is caused by intense depolarization of neurons and astrocytes, propagating along the cortical gray matter at a speed of 2–6 mm per minute (Leao, 1944). As it spreads, it depresses the ongoing electrical activity (e.g., the EEG as originally observed), hence, named spreading depression by Leao. However, cortical spreading depolarization is increasingly used instead of cortical spreading depression because of the confusion the term “depression” causes. Leao was the first to note the similarity between the propagation rate of visual aura in migraine and CSD (Leao, 1947). However, it only became possible to show the presence of CSD in migraineur brain in the past 4 decades with advances in imaging technologies that enabled detection of the CSD-induced cerebral blood flow changes (Olesen et al., 1982; Cao et al., 1999; Hadjikhani et al., 2001).

High extracellular levels of glutamate and K^+^ are thought to be responsible for ignition and propagation of CSD (Pietrobon and Moskowitz, 2014). Rising extracellular glutamate levels along with K^+^ concentration exceeding 15 mM appear to be necessary for synchronized depolarization of an aggregate of neurons involving, at least partly, the NR2 type NMDA receptors (Tang et al., 2014; Takizawa et al., 2020a; Bu et al., 2020). During CSD, K^+^ levels further rise to 30–60 mM, which contribute to spread of the depolarizing wave to neighboring gray matter (Pietrobon and Moskowitz, 2014). Accordingly, application of glutamate, NMDA or high K^+^ to the cortex all ignites CSD. Therefore, increased glutamate release in FHM1 knock-in mice is consistent with the enhanced susceptibility to CSD (Pietrobon and Brennan, 2019). Indeed, foci of glutamate “plumes” spontaneously bursting in the cortex of awake *ATP1A2* knock-in mice, a model of FHM2, have recently been demonstrated by expressing a fluorescent glutamate reporter in the cortex (Parker et al., 2021). The same study also showed that a surge of glutamate plumes preceded the onset of CSD as hypothesized before.

Astrocytic Na/K pump (α2 Na/K-ATPase) clears K^+^ spilling out of the synaptic cleft during synaptic activity (Cholet et al., 2002). The transmembrane Na^+^ gradient it creates as it takes up K^+^ is essential for uptake of glutamate by astrocyte processes around excitatory synapses (Cholet et al., 2001; Petit et al., 2021). Accordingly, ATP1A2 hypofunction caused by FHM2 mutations leads to reduced K^+^ and glutamate uptake as demonstrated both *in vitro* and in knock-in mice, *in vivo* (Capuani et al., 2016; Parker et al., 2021). As with *CACNA1A* mutations, GABA release is not affected in FHM2 because α2 Na/K-ATPase is not appreciably expressed on astrocyte processes around GABAergic terminals (Cholet et al., 2002; Capuani et al., 2016). Consistent with these findings, FHM2 knock-in mice have a low CSD induction threshold and spontaneously generate the glutamate plums mentioned above (Leo et al., 2011; Parker et al., 2021).

Although the pathophysiological phenotype for FHM1 and FHM2 converge on the glutamatergic synapse, this is not the case for *SCN1A* gene mutations underlying FHM3 (Desroches et al., 2019; Chever et al., 2021; Lemaire et al., 2021). *SCN1A* encodes the α1 subunit of the neuronal voltage-gated sodium channel Nav1.1, which contributes to the action potential firing on primarily GABAergic interneurons. Indeed, hundreds of loss-of-function *SCN1A* mutations have been reported in various epileptic syndromes caused by reduced GABAergic inhibition (Hedrich et al., 2014; Sutherland HG. et al., 2019; Mantegazza et al., 2021). In FHM3, however, mutations are gain-of-function, causing increased firing of GABAergic interneurons. Increased GABAergic activity may sound at odds with CSD generation but recent studies have shed light on the mechanism of CSD initiation by documenting that hyperactive GABAergic interneurons discharging at high frequencies can cause significant elevations in extracellular K^+^ (over 12 mM), hence, increase CSD susceptibility as discussed above (Desroches et al., 2019; Chever et al., 2021; Lemaire et al., 2021). Supporting this view, knock-in mice bearing *L263V* or *L1649Q* human mutation exhibit a low CSD induction threshold (Jansen et al., 2020; Auffenberg et al., 2021) as well as spontaneous CSDs (Jansen et al., 2020) and hyperexcitable GABAergic neurons (Auffenberg et al., 2021).

Another gain of function change in glutamatergic transmission secondary to impairment of frequency-dependent presynaptic adaptation has been reported for mutations in casein kinase 1 delta, which cause a non-hemiplegic form of familial migraine along with disrupted sleep phases (Brennan et al., 2013). Knock-in mice harboring this mutation have also a reduced CSD induction threshold (Suryavanshi et al., 2019). Knock-in mouse models have been instrumental to study various aspects of migraine although they basically model familial migraines. In addition to enhanced susceptibility to CSD, these mice also exhibit heightened sensory perceptions such as hypersensitivity to light, consistent with sensory abnormalities reported by patients (Russell and Ducros, 2011; Chanda et al., 2013; Brennan and Pietrobon, 2018). They also seem to suffer from headaches and periorbital allodynia (Langford et al., 2010; Chanda et al., 2013). Mutations associated with severe clinical phenotypes additionally lead to spontaneous seizures or ataxia in FHM 1 and 2 knock-in mice (Van Den Maagdenberg et al., 2010; Kros et al., 2018).

### Non-Familial Common Migraine

The mutations detected in familial migraine are usually not found in non-familial common migraine cases although migraine has been established to have a complex polygenic genetic heritability estimated to be as high as 60% (Sutherland H. G. et al., 2019). Meta analysis of several GWAS studies encompassing 59,674 patients and 316,078 controls reveled 44 susceptibility loci (Gormley et al., 2016). Most of them are associated with vascular and neuronal function, ion channels and circadian rhythm (Sutherland H. G. et al., 2019). Three of them are near ion channels (TRPM8 and KCNK5) or an ion transporter (sodium/potassium/calcium exchanger3 SLC24A3). TRPM8 is expressed on type C- and A-delta nociceptors and is activated by cold temperatures (Dussor and Cao, 2016). Although migraine is generally initiated by intrinsic brain mechanisms, it can also be triggered by some volatile irritants such as umbellulone emanating from the leaves of headache tree (*umbellularia californica*), which is thought to cause headache by stimulating TRPA1 channels on meningeal nociceptors (Edelmayer et al., 2012). KCNK5 belongs to the superfamily of potassium channel proteins containing two pore-forming P domains as does KCNK18 (Reyes et al., 1998). Although the neuronal function of KCNK5 is unclear, TRESK potassium channels encoded by *KCNK18* regulates neuronal excitability in the dorsal root and trigeminal ganglia (Enyedi and Czirjak, 2015). Supporting a role for two-pore-domain potassium channels in migraine, *KCNK18* mutations cause non-hemiplegic familial migraine with aura. F139Wfsx24 *KCNK18* frame-shift mutation has been shown to result in hyperexcitability of cultured trigeminal ganglion neurons as well as allodynia in rodent migraine models (isosorbide dinitrate injection) by a negative dominant effect on TREK1 and 2, two other two-pore-domain potassium channels with which TRESK channels form functional heterodimers (Liu et al., 2013; Royal et al., 2019). Suggesting a suppressive role of TRESK over trigeminal ganglia excitability, KCNK18 knockout mice reportedly exhibit mechanical and thermal hyperalgesia in response to systemic glyceryl trinitrate treatment (a migraine model) (Pettingill et al., 2019) and exaggerated nocifensive behaviors in response to dural application of inflammatory soup (Guo et al., 2019). Of note, these families with KCNK18 mutations exhibit migraine with aura, suggesting that reduced control over neuronal hyperexcitability due to hypofunction of TRESK channels in cortical neurons may also create susceptibility to CSD.

### Initiation of Inflammatory Signaling That Causes Headache

CSD has been shown to activate neuronal pannexin1 channels and initiate the downstream inflammatory signaling following formation of the inflammasome complex (Karatas et al., 2013; Ghaemi et al., 2016; Takizawa et al., 2016; Chen et al., 2017; Eising et al., 2017; Ghaemi et al., 2018; Bu et al., 2020; Takizawa et al., 2020b). Inflammasome formation causes release of pro-inflammatory mediators such IL1-ß and HMGB1 from neurons, which triggers translocation of inflammatory transcription factor NF-kappaB to the nucleus in astrocytes (Karatas et al., 2013; Takizawa et al., 2016; Dehghani et al., 2021). NF-kappaB induces hundreds of transcripts including tens of inflammatory mediators such as iNOS, COX2, and cytokines (https://www.bu.edu/nf-kb/gene-resources/target-genes/). This is thought to cause release of prostanoids, NO, cytokines and other algesic mediators to CSF and, hence, to activate meningeal nociceptors and inflammatory cells, culminating in a sterile meningeal inflammation that can sustain migraine headache for hours to days (Karatas et al., 2013; Erdener and Dalkara, 2014; Dalkara and Moskowitz, 2017). With a single CSD, microglia do not switch to a pro-inflammatory state, which is only seen a few days after exposure to multiple CSDs (Takizawa et al., 2017), suggesting that aura (CSD)-triggered parenchymal inflammatory signaling is mainly mediated by astrocytes, whereas microglia may take part in inflammatory signaling in patients suffering from frequent migraine with aura attacks. Supporting this formulation, a recent PET/MRI study using a sensitive inflammatory tracer (^11^C-PBR28) taken up by active glia, has documented the presence of parenchymal as well as meningeal inflammatory signaling in 13 patients suffering from repeated migraine with aura attacks in the past month (Albrecht et al., 2019; Hadjikhani et al., 2020).

Interestingly, in the absence CSD, it has been shown that parenchymal inflammatory signaling pathway can be initiated via activation of neuronal Panx1 channels, this time, by migraine triggers such as sleep deprivation (Kilic et al., 2018). Sleep deprivation induces transcriptional changes favoring glycogen synthesis over its breakdown (Petit and Magistretti, 2016; Petit et al., 2021). Insufficient glycogen breakdown within astrocyte processes around glutamatergic terminals can lead to Panx1 channel activation due to inadequate glutamate and K^+^ clearance. This is because glycogen-derived ATP is preferentially used for glutamate and K^+^ uptake even in the presence of sufficient glucose owing to more favorable kinetics of the enzymes involved in metabolizing the glycosyl units liberated from glycogen over glucose, whereas glucose is quickly metabolized to replenish glycogen (Petit et al., 2021). Thus, extracellular glutamate and K^+^ accumulation during intense glutamatergic synaptic activity create a favorable extracellular milieu, first, for activation of Panx1 channels, and then, for CSD ignition upon further rise in extracellular glutamate and K^+^ (Kilic et al., 2018; Petit et al., 2021). This proposed mechanism could explain why migraine with and without aura attacks exist in the same person and how CSD can emerge in the absence of monogenic mutations but with transcriptional changes triggering the same mechanisms in non-familial migraine.

### Master Switch of Inflammatory Signaling: Pannexin Channels

**FIGURE 2:**
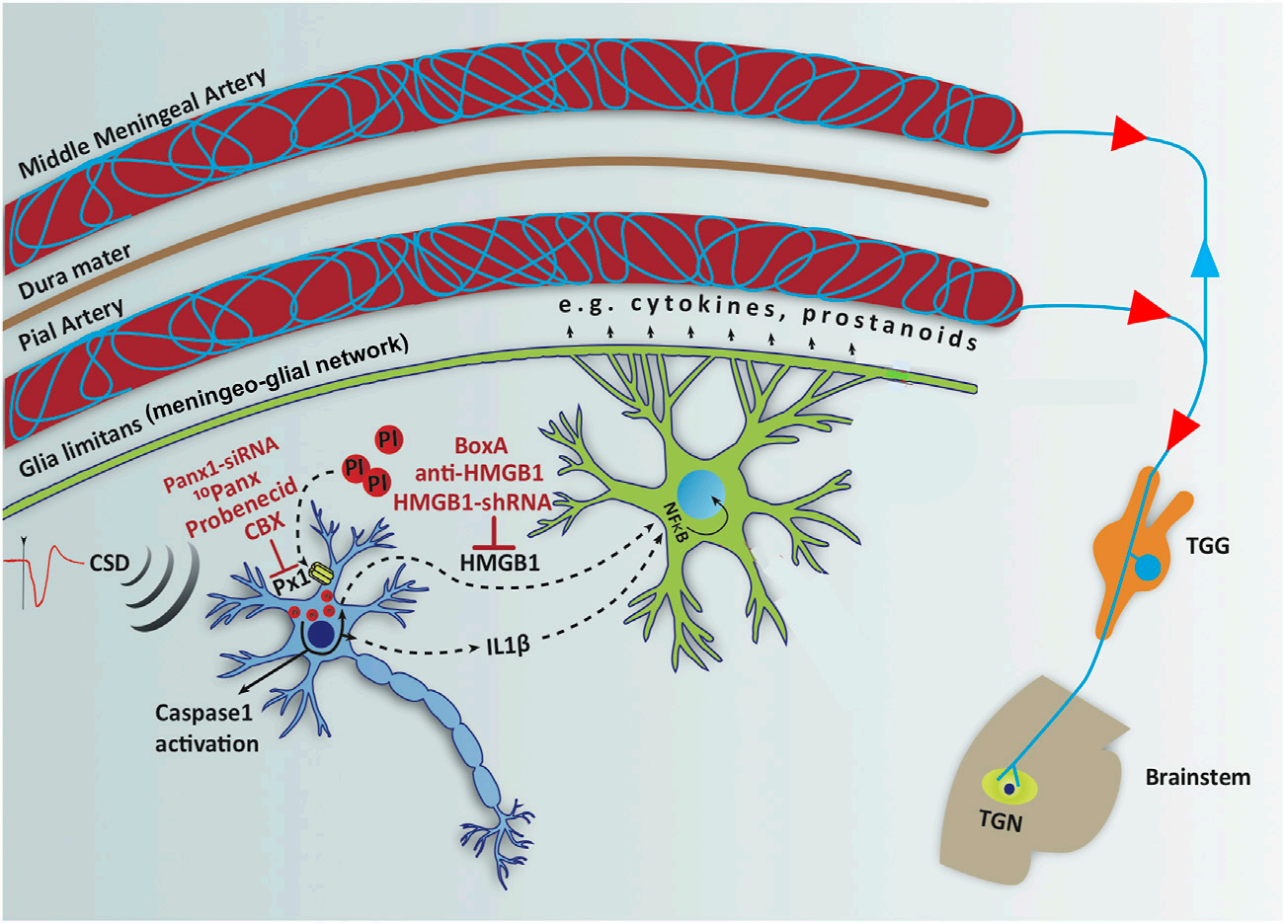
Opening neuronal Panx1 channels after CSD initiates a neuroinflammatory signaling cascade, characterized by inflammasome formation and caspase-1 activation in neurons followed by release of HMGB1 and IL-1β, which trigger NF-κB nuclear translocation in astrocytes. Red labels show agents used to inhibit each step in the inflammatory cascade and red circles represent propidium iodide (PI) influx through open Panx1 (Px1) large-pore channels. NF-κB induces transcription of several pro-inflammatory genes including cytokines and prostanoids, which are thought to be released to CSF through glia limitans and meningeo-glial network and activate the nociceptive nerves around pial and dural vessels, causing headache. Modified from Karatas et al., 2013 with permission.

Pannexins are large-pore membrane channels similar to connexins but, unlike connexins, they do not form gap junctions (Macvicar and Thompson, 2010). Panx1 and Panx2 are widely expressed in the central nervous system on all main cell types (Yeung et al., 2020). *In vitro* studies have disclosed that Panx1 can open in three different states; a low conductance Cl-selective opening at positive membrane potentials (*in vivo* function unknown); large conductance, non-selective opening allowing calcium, ATP and molecules up to 900 Dalton to pass through; and a persistent opening due to caspase-mediated cleavage of the C-terminal domain, leading to cell death (Dahl, 2018). Panx1 channels can be physiologically activated in large-pore state by increases in extracellular K^+^, glutamate and intracellular Ca^2+^ concentration, NMDA and P2X7 receptor stimulation, swelling (membrane stretch), c-Jun N-terminal kinases and Src family of tyrosine kinases (Whyte-Fagundes and Zoidl, 2018). Most of the Panx1 activating conditions are present during CSD as well as when glycogen breakdown, hence, K^+^ and glutamate uptake is reduced as discussed above (Karatas et al., 2013; Kilic et al., 2018; Petit et al., 2021). Opening of neuronal Panx 1 channels can be monitored *in vivo* and *in vitro* by using membrane-impermeant fluorescent dyes like propidium iodide or YoPro-1 (Macvicar and Thompson, 2010), which flux into cells through Panx 1 channels opened in large conductance state (Karatas et al., 2013; Bu et al., 2020) (Figure 2). CSD-induced dye uptake to neurons was suppressed with several Panx1 inhibitors such as carbenoxolone, probenecid, ^10^Panx peptide and inhibition of Panx1 expression by siRNA, supporting the view that the dye fluxed in neurons through Panx1 channels (Karatas et al., 2013; Bu et al., 2020). Large pore opening of P2X7 receptors can also mediate the dye influx (Bhaskaracharya et al., 2014). However, recent transcriptomics studies consistently show that P2X7 receptors are not expressed in adult neurons unlike macrophages where they are closely coupled with Panx1 channels (Illes et al., 2017; Kaczmarek-Hajek et al., 2018). Further supporting opening of Panx1 channels with CSD or insufficient glycogen breakdown, formation of the inflammasome complex and activation of caspase-1 along with HMGB1 release have also been demonstrated (Karatas et al., 2013; Takizawa et al., 2016; Kilic et al., 2018). These advances open the exciting possibility of developing Panx1 inhibitors as migraine prophylactic drugs; especially considering some Panx1 inhibitors such as carbenoxolone, probenecid and mefloquine are already clinically registered medicines (Yeung et al., 2020). Similarly, agents acting on downstream steps in the inflammatory cascade including inhibitors of inflammasome, caspase-1 or interleukins may be promising drug targets for migraine treatment. Successes of anakinra in relieving severe migraine headaches seen in cyropyrin-associated periodic syndromes characterized by IL1-ß over production due to inflammasome mutations (Parker et al., 2016) and, the relief obtained by NSAIDs in treating common migraine headaches support this view.

## Ion Channel Dysfunctions in Stress and Depression

As proposed for migraine, changes in the excitation/inhibition (E: I) balance resulting in increased excitability have also been implicated in major depression (Fee et al., 2017). Ion channels are important regulators of excitability, network activity as well as plasticity. Changes in ion channel attributes alter GABAergic and glutamatergic neuron excitability and firing, hence, modify the E: I balance in microcircuits. A recent transcriptomic analysis revealed that out of 1,153 differentially expressed genes (DEGs) in the hippocampi of chronically stressed rats, a rodent model of depression; 46 DEGs were related to potassium channels, calcium channels, sodium channels, and chloride channels on plasma membrane (Ren et al., 2021). This finding supports the hypothesis that changes in the expression or function of ion channels may disrupt the E: I balance throughout the brain regions relevant for depression, and contribute to the development of depression. Indeed, a recent paper reported that global inactivation of γ2 subunit gene of GABA-A receptors on somatostatin interneurons (SST-IN) disinhibited effects of SST-IN on pyramidal cells, resulting in an anxiolytic and antidepressant phenotype (Fuchs et al., 2017). On the other hand, ionotropic P2X7 receptors can additionally trigger formation of the inflammasome complex, activating interleukin-1ß and downstream inflammatory signaling, as do Panx1 channels. Indeed, several lines of evidence implicate the involvement of P2X7 receptors and inflammatory signaling, including inflammasome formation in the pathophysiology of stress and depression (Dowlati et al., 2010; Iwata et al., 2016; Wohleb et al., 2016; Yue et al., 2017). Accordingly, in the second part of the article, we will review the potential roles of purinergic P2X7 receptors along with hyperpolarization-activated cyclic nucleotide-gated (HCN) channels, M-type K^+^ (KCNQ) channels, TWIK-related K^+^ channels (TREK) and Ca^+2^ channels in the pathophysiology of depression, dysfunction of which are also implicated in migraine.

### P2X7 Receptor

The purinergic P2X7 receptor (P2X7R) is a ligand gated ion channel activated by binding of adenosine triphosphate (ATP) (Khakh and North, 2006). ATP is released from the presynaptic terminal as a co-transmitter as well as from astrocytes (Bezzi and Volterra, 2001; Burnstock, 2007). Its release is increased in response to physical stressors and cellular injury as part of an adaptive response. Psychological stressors like acute restraint stress, in which restriction of the movement of animals leads to stress, also have been shown to increase extracellular ATP concentrations in rat hippocampus and prefrontal cortex (PFC) (Iwata et al., 2016), which then can activate low affinity P2X7R (Donnelly-Roberts et al., 2009). Mice susceptible to chronic social defeat stress (CSDS), on the other hand, displayed lower ATP concentrations in hippocampus and PFC (Cao et al., 2013). In line with the latter finding, intracerebroventircular administration of ATP or its nonhydrolyzable analog, ATP-γ-S, for 7-days or 28-days reverses social avoidance caused by CSDS and anhedonia caused by chronic unpredictable mild stress (CUMS) (Cao et al., 2013) (Table 1). Inhibition of ATP release selectively from astrocytes by genetic knockout of inositol 1,4,5- trisphosphate (IP3) receptor type 2 (*IP3R2* ^−/−^) lead to increased behavioral despair and anhedonia (Cao et al., 2013) (Table 1). These findings suggest that increased ATP release from astrocytes in the hippocampus and PFC in response to stressors is an important mediator in developing adaptive responses to stress.

**TABLE 1:** Behavioral effects of interventions to P2X7R expression and functions.

Genetic interventions	Species	Results	References
P2RX7^−/−^	C57BL/6 mice	Decreased immobility time in TST and FST.Reduced anhedonia in SPT after stimulation with a bacterial endotoxin, lipopolysaccharide	Basso et al., (2009),
			Csolle et al. (2013a),
			Csolle et al. (2013b)
*hP2X7R/hP2RX7-Gln460Arg* homozygotes and heterozygotes	C57BL/6 mice	Basal levels of depressive-like behavior were unaltered. CSDS induced increased social avoidance, decreased time spent in open arms in EPM in all genotypes studied.	Metzger et al. (2017)
*hP2RX7-WT-hP2RX7-Gln460Arg* heterozygotes	C57BL/6 mice	Decreased slow wave activity&NREM sleep duration and increased number of REM sleep bouts	Metzger et al. (2017)
*IP3R2* ^−/−^	C57BL/6 mice	Selective inhibition of astrocytic ATP release lead to increased behavioral despair and anhedonia	Cao et al. (2013)
P2X7R AGONISTS			
ATP/ATP-γ-S (icv)	C57BL/6J mice	Reversed social avoidance caused by CSDS and anhedonia caused by CUMS	Cao et al. (2013)
ATP/ATP-γ-S (intra mPFC)		Reversed the increase in immobility time in FST	
ATP/BzATP (intrahippocampal, 3 weeks)	SD rats	Increased immobility time in FST	Yue et al. (2017)
P2X7R ANTAGONISTS			
BBG/A438079 (3 weeks, intrahippocampal, with CUS)	SD rats	Reversed increased immobility caused by CUS	Yue et al. (2017)
A-804598 (4 weeks, ip with CUS)	SD rats	Reversed CUS-induced deficits in SPT, NSFT and EPM	Iwata et al. (2016)
Brilliant Blue G (1 week, ip)	C57BL/6 mice	Decreased immobility time in TST	Csölle et al. (2013a)
PPADS into mPFC	C57BL/6 mice	Blocked the antidepressant-like effect of ATP on immobility time in FST	Cao et al. (2013)

ATP, adenosine triphosphate; CSDS, chronic social defeat stress; CUMS, chronic unpredictable mild stress; CUS, chronic unpredictable stress; EPM, elevated plus maze; FST, forced swim test; icv, intracerebroventricular; *IP3R2,* inositol 1,4,5- trisphosphate receptor type 2; ip, intraperitoneal; mPFC, medial prefrontal cortex; NSFT, novelty suppressed feeding test; SD, Sprague-Dawley; SPT, sucrose preference test; TST, tail suspension test.

**FIGURE 3:**
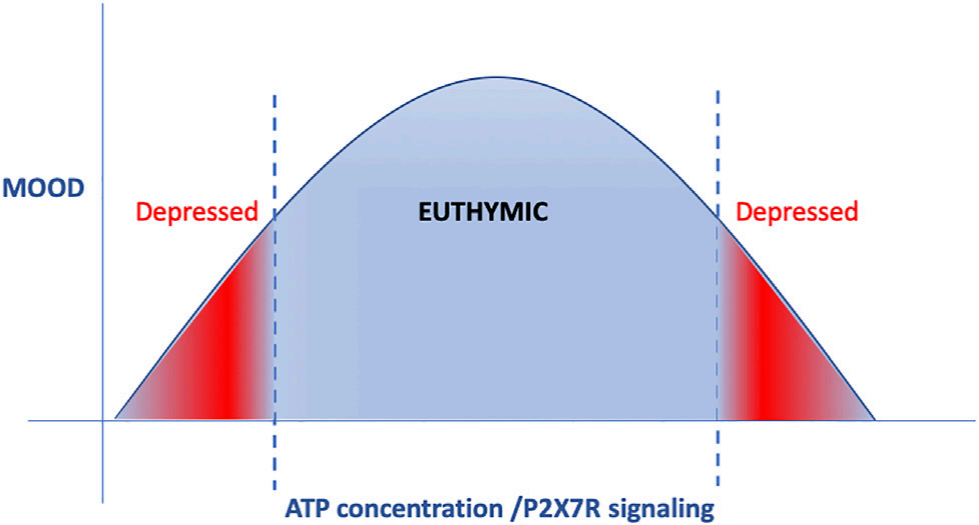
ATP and P2XR signaling displays a reverse U-curve. Both very low and very high doses of ATP and P2XR signaling are involved in the development of depression-like behavior, whereas moderate doses are necessary to maintain euthymic mood.

However, the story is more complex: Intra-PFC, but not intrahippocampal injections of ATP or ATP-γ-S reversed the depression-like behavior (Cao et al., 2013); whereas chronic (3weeks) intrahippocampal injections of ATP or BzATP, a P2XR agonist, induced depression-like behavior to a similar extent as chronic unpredictable stress (CUS) (Table 1). Long-term blockade of P2X7R for 3–4 weeks by systemic (intraperitoneal, i.p.) or intrahippocampal injections of selective P2XR7 antagonists Brilliant Blue G (BBG), A438079, A-804598, reversed depressogenic behavior, specifically behavioral despair, anhedonia and anxiety-like behavior caused by CUMS in rats (Iwata et al., 2016; Yue et al., 2017) (Table 1). Subacute (1 week) but not acute systemic treatment of mice with BBG also caused a reduction in behavioral despair (Csolle et al., 2013a) (Table 1). Further complicating understanding the role of P2X7R in depression, preinfusions of a non-selective P2XR antagonist, pyridoxal phosphate-6-azophenyl-2′-4′-disulphonic acid, and P2X2R shRNA into the mPFC blocked the antidepressant-like effect of ATP (Cao et al., 2013) (Table 1). These findings altogether suggest that; 1) ATP and P2XR signaling exhibit an inverted U-shaped relationship in mood regulation; i.e., both very low and very high doses of ATP and P2XR signaling are involved in the development of depression-like behavior whereas moderate doses are necessary to maintain euthymia (Figure 3); 2) effects of ATP and P2XR signaling are region-specific.

Genetic tools, in which control of the “dose effect” is far limited compared to pharmacological approaches, have confirmed the important role of P2XR signaling in the pathophysiology of depression but the net consequnce of their effect was also model-dependent. Studies using *P2RX7* ^−/−^ KO mice reported a decrease in behavioral despair, i.e. an antidepressant effect (Basso et al., 2009; Csolle et al., 2013a; Csolle et al., 2013b) (Table 1). In human genetic studies, polymorphisms of *P2RX7* the gene encoding P2X7R has been reported to be associated with increased risk/severity of mood disorders (Lucae et al., 2006; Mcquillin et al., 2009; Halmai et al., 2013; Vereczkei et al., 2019). As a more precise model of the human condition, a humanized transgenic mouse line was generated in which exon 2–13 of murine P2X7R was substituted by either human wild-type (WT) *hP2X7R* or *hP2X7R-Gln460Arg* variant. Intriguingly, the ion channel function of *hP2X7R-Gln460Arg* variant was not different than that of the WT, but it was impaired when *hP2X7R-Gln460Arg* variant was co-expressed with WT P2X7R (Aprile-Garcia et al., 2016). Contrary to *P2x7R* KO mice, mice homozygous or heterozygous for *hP2X7R-Gln460Arg* allele displayed no change in depression-like behavior at basal conditions, but they showed increased depression- and anxiety-like behavior following social defeat stress (Metzger et al., 2017) (Table 1). These findings indicate that *P2X7R* polymorphism creates vulnerability to stress and depression and supports the involvement of gene X environment interactions in the development of mood disorders. Interestingly sleep quality was disturbed in heterozygous mice (*WT hP2X7R/hP2X7R-Gln460Arg*), but not in homozygous mice (*hP2X7R-Gln460Arg/hP2X7R-Gln460Arg*) (Metzger et al., 2017). Specifically, slow wave activity and NREM sleep duration were reduced and the number of REM sleep bouts was increased in heterozygous mice (Metzger et al., 2017) (Table 1), which is similar to sleep disturbances seen in depressive patients (Nutt et al., 2008). These findings have led to a randomized, placebo-controlled, double blinded clinical trial in order to evaluate the antidepressant efficacy of P2X7R antagonists in the treatment of major depression (ClinicalTrials.gov Identifier: NCT04116606).

### Potential Mechanisms Underlying P2X7R’s Effects on Mood Regulation

P2X7R mediates several physiological neural functions, disturbances of which may be involved in the pathophysiology of depression:

1. P2X7R increases the release of neurotransmitters noradrenaline (NA), serotonin (5-HT), glutamate and GABA (Sperlagh et al., 2002; Papp et al., 2004; Alloisio et al., 2008; Marcoli et al., 2008; Goloncser et al., 2017). These neurotransmitters long implicated in the pathophysiology of depression also modulate astrocytic ATP release by altering intracellular Ca^+2^ levels (Hirschfeld, 2000; Arcuino et al., 2002; Luscher et al., 2011; Marpegan et al., 2011; Duman et al., 2019; Fogaca and Duman, 2019). Indeed, selective inhibition of ATP release from astrocytes by genetic knockout of IP3R2 mediating intra-astrocytic Ca^+2^ rise induces depression-like behavior (Cao et al., 2013), which may be related with the resulting dysregulation of the release of neurotransmitters.

2. P2X7R regulates synaptic plasticity. Chronic stress has been reported to decrease synaptic plasticity, which is thought to be involved in depression-like phenotype by limiting adaptive responses. In *P2RX7* ^−/−^ KO mice, whole genome microarray analysis disclosed changes in expression of genes involved in synaptic plasticity (Csolle et al., 2013a). P2X7R ligand ATP has been shown to alter synaptic plasticity by several mechanisms, including glutamate release and regulation of NMDA receptor expression and function (Pankratov et al., 2002; Sperlagh et al., 2002; Csolle et al., 2013b; Guo et al., 2015; Otrokocsi et al., 2017). ATP and BzATP application evoked glutamate efflux in hippocampal slices obtained from WT mice, which was abolished in hippocampal slices of *P2RX7* ^−/−^ mice (Sperlagh et al., 2002; Csolle et al., 2013b). There is an ongoing debate on the presence of P2X7R in adult neurons (Illes et al., 2017; Miras-Portugal et al., 2017). P2X7R was proposed to modulate presynaptic release of glutamate, however, recent transcriptomic studies report that neurons isolated from adult rodents do not express P2X7R unlike the *in vitro* preparations prepared from young or embryonic animals (Illes et al., 2017; Kaczmarek-Hajek et al., 2018). Therefore, the glutamate efflux induced by ATP and P2X7R agonists in adult animals can also be attributed to the astrocytic release (Parpura et al., 1994; Savtchouk and Volterra, 2018). The basal and stress-induced expression of the NR2B subunit of NMDA receptor was found to be upregulated in *P2RX7* ^−/−^ mice both *in vitro* and *in vivo* (Csolle et al., 2013b; Otrokocsi et al., 2017), possibly as a compensatory response to decreased glutamate release. As P2X7R ion channel has considerable Ca^+2^ permeability, when expressed at the synapse, P2X7R can modulate synaptic plasticity. Indeed, in hippocampal slices prepared from 3-week old rats, ATP blocked induction of long-term potentiation (LTP) with low frequency stimulation at the CA3-CA1 synapse by inhibiting NMDA-mediated currents through Ca^+2^-dependent inactivation of NMDA receptors. At higher frequencies of stimulation, owing to desensitization of P2X receptors, ATP loses its inhibitory effect on LTP (Pankratov et al., 2002). In accordance with these observations inhibition or desensitization of P2XR with pyridoxal phosphate-6-azophenyl-2′-4′-disulphonic acid or nonhydrolyzable ATP analog α,β-methylene ATP facilitated LTP at the CA3-CA1 synapse (Pankratov et al., 2002).

3. P2X7R can induce neuroinflammation. Binding of ATP to P2X7R trigger NLRP3 inflammasome cascade resulting in the activation of caspase-1, which converts pro-IL-1ß and pro-IL-18 to their active forms: IL-1ß and IL18 (Guo et al., 2015). As mentioned above, psychological stressors increase extracellular ATP levels, which reportedly lead to activation of caspase-1 and IL-1ß release by binding to P2X7R on microglia (Iwata et al., 2016; Yue et al., 2017). This may be related to the increased peripheral levels of cytokines, acute phase proteins and chemokines detected in depressed patients (Levine et al., 1999; Lanquillon et al., 2000; Alesci et al., 2005; Pace et al., 2006; Dowlati et al., 2010; Milenkovic et al., 2019). Indeed, increased microglial activity in the prefrontal cortex, anterior cingulate cortex and insula of major depression patients was visualized by positron emission tomography (Setiawan et al., 2015).

The role of microglia in sculpturing synapses is well documented. Microglial processes in close contact with dendritic spines can function in both the elimination and formation of spines, suggesting that they play important roles in synaptic plasticity (Paolicelli et al., 2011; Zhan et al., 2014; Miyamoto et al., 2016; Weinhard et al., 2018; Cangalaya et al., 2020). Stress is known to cause a decrease in dendritic length and branching in apical dendrites of layer II/III and layer V pyramidal neurons of the medial prefrontal cortex as well as apical dendrites of CA3 pyramidal neurons (Mcewen, 1999; Mcewen, 2001; Cook and Wellman, 2004; Liston et al., 2006; Radley et al., 2006; Czeh et al., 2008; Goldwater et al., 2009). On the other hand, chronic stress increases the length of apical dendrites in the ventral orbital subregion of the orbitofrontal cortex as well as the dendritic arborization and spine density in the basolateral amygdala spiny neurons (Vyas et al., 2003; Dias-Ferreira et al., 2009). Recent work showed that microglia activated after chronic unpredictable stress were involved in pruning of spines both in the Layer-1 of mPFC and hippocampus (Milior et al., 2016; Wohleb et al., 2018). Interestingly, deletion of P2X7R gene inhibited the decrease in synapse numbers in dorsal dentate gyrus (DG) following inescapable footshock, a rodent model of depression (Otrokocsi et al., 2017), indicating a possible role of P2X7R in pruning of synapses, which needs to be addressed in future studies.

In conclusion, as with ATP release, ATP-P2X-mediated microglial activation may exert depressant or anti-depressant effect possibly depending on the intensity of the stimulus and brain region, which may yield contrasting results in *in vitro* and *in vivo* experiments. Further studies are needed to document the prevailing mechanisms involved under *in situ* conditions as well as in patients.

### Hyperpolarization-Activated Cyclic Nucleotide-gated Channels and M-type K^+^ (KCNQ) Channels

Hyperpolarization-activated cyclic nucleotide-gated (HCN) channels are nonselective cation channels located mainly at dendrites, but also present at axons and the neuronal soma. They open at membrane voltages more negative than −40 mV and when HCN channels open, membrane resistance decreases (Biel et al., 2009; Kase and Imoto, 2012). Thus, HCN channels can counteract both membrane hyperpolarization and depolarization allowing both neuronal excitation as well as inhibition (Kase and Imoto, 2012; Benarroch, 2013). Four subtypes of HCN’s have been identified: HCN1 to HCN4. HCN1 and HCN2 were reported to be highly expressed in brain regions known to be involved in the pathophysiology of major depression, namely prefrontal cortex, hippocampus, ventral tegmental area (VTA) and nucleus accumbens (NAc) (Notomi and Shigemoto, 2004).

HCN’s interact with cyclic adenosine monophosphate (cAMP), which modulates voltage dependence of their activation kinetics and facilitate their opening. Signaling through several neuromodulators like noradrenaline and dopamine, whose dysfunction may contribute to the pathophysiology of depression, alter cAMP levels in the dendrites and thus HCN channel functioning, especially of HCN 2, the subtype most strongly modulated by cAMP (Biel et al., 2009). HCN also interacts with tetratricopeptide repeat-containing Rab8b interacting protein (TRIP8b), which regulates trafficking of HCN to dendrites (Biel et al., 2009).

**FIGURE 4:**
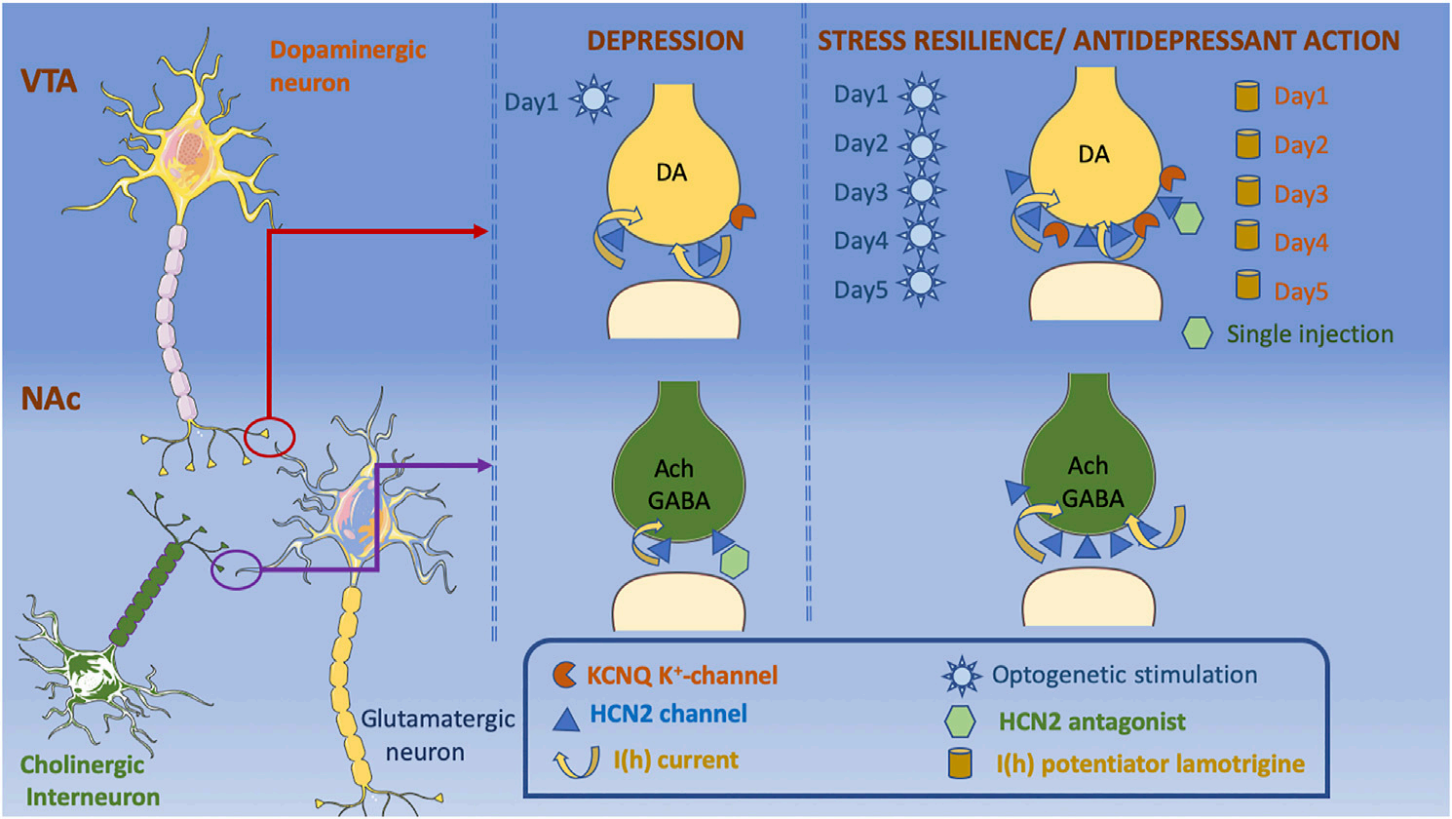
The decrease in the expression of HCN2 channels in NAc on both dopaminergic terminals projecting from VTA and local ChIN terminals is depressogenic. Decrease in I(h) currents in ChIN terminals is associated with depression-like phenotype, whereas an increase in I(h) currents on the VTA dopaminergic terminals is observed in both stress susceptible and stress resilient mice. K^+^ channels are increased only in the stress resilient mice counterbalancing the effects of increased I(h) currents. Application of an HCN2 antagonist into NAc causes a depressogenic phenotype, whereas it has antidepressant effects when applied into VTA. Lamotrigine administration for 5-days also reduce depressogenic behaviors. Acute optogenetic stimulation of VTA dopaminergic neurons increase depression-like behaviors, whereas chronic optogenetic stimulation of VTA dopaminergic neurons shows antidepressant effects. Cellular templates are copied from Servier Medical Art (smart.servier.com).

Global knockout of *HCN1*, *HCN2,* or *TRIP8b* genes (leading to a decrease in both HCN1 and HCN2 protein levels) in mice resulted in a reduction in behavioral despair, an antidepressant-like effect (Lewis et al., 2011) (Table 2). Studies showed that HCNs regulate neuronal excitability and plasticity in response to stress, depending on the brain region and cell type that they are expressed in, as well as the duration and type of the stressor. For example, VTA dopamine (DA) neurons in the brain reward circuit has important role in regulation of the stress response. Evidence indicates that there are disturbances in the brain reward circuitry in major depression, which is associated with anhedonia, one of the 2 cardinal symptoms of major depression (Nestler and Carlezon, 2006; Russo and Nestler, 2013; Nestler, 2015; Hoflich et al., 2019). Recent evidence showed that the social stress susceptible mice display increased VTA DA neuronal firing and increase in HCN mediated I(h) currents (Cao et al., 2010; Chaudhury et al., 2013), whereas mice exposed to chronic unpredictable mild stress show a reduction in I(h) and DA neuronal firing (Zhong et al., 2018) (Table 2). In NAc, one of the major targets of VTA DA neurons, both HCN 2 expression and function [I(h)] were reduced in cholinergic interneurons (ChIN) in two different mice depression models, namely chronic social defeat stress and *p11* knockout mice (Cheng et al., 2019) (Figure 4). Because decreased activity of ChIN in NAc was found to be related to depressive behavior, the possible involvement of HCN2 channels in regulating ChIN activity was studied by pharmacological and genetic interventions. Decreased tonic firing of ChIN by pharmacological blockade of HCN2 channels (ZD7288) suggest that dysfunction of HCN channels participates in the diminished ChIN firing rate observed in depressive mice (Table 2; Figure 4). Intriguingly, selective overexpression of HCN2 in both NAc ChIN and VTA DA neurons resulted in antidepressant-like effects (Friedman et al., 2014; Zhong et al., 2018; Cheng et al., 2019) (Table 2; Figure 4). Selective knockdown of HCN2 in VTA DA neurons by shRNA increased anxiety-like and depression-like behaviors (Zhong et al., 2018). Intriguingly, knockdown of HCN1 in CA1 region of dorsal hippocampus resulted in anxiolytic and antidepressant effects (Kim et al., 2012) (Table 2). Whether this effect was associated with the difference in HCN subtype or the brain region studied warrants further research investigating the role of HCN channels in other brain regions relevant to depression.

**TABLE 2:** Effects of modulating HCN2 expression and function on depression-like behavior.

HCN channels			
Intervention	Affected brain region	Results	References
CUMS	VTA	Decreased DA neuronal firing and I(h)	Zhong et al. (2018)
		Increased depression-like behavior	
CSDS	VTA	Increased DA neuronal firing and I(h)	Cao et al. (2010)
		Increased depression-like behavior	Chaudhury et al. (2013)
CSDS	NAc	Decreased HCN 2 expression and I(h) in ChIN	Cheng et al. (2019)
*P11* ^−/−^		Increased depression-like behavior	
*HCN1* ^−/−^	Global knockout	Decreased time spent immobile in TST and FST; increased social interaction	Lewis et al. (2011)
*HCN2* ^−/−^			
*TRIP8b* ^−/−^			
HCN2 antagonist			
ZD7288 (on NAc slices)	NAc	Decrease ChIN firing	Cheng et al. (2019)
ZD7288 (into VTA)	VTA	Reverse CSDS-induced social avoidance	Cao et al. (2010)
DK-AH 269 (into VTA)			
I(h) potentiator			
Lamotrigine (into VTA, for 5 days)	VTA	Reversed CSDS-induced social avoidance and decrease in sucrose preference	Friedman et al. (2014)
HCN2 overexpression	NAc ChIN	Reversed social avoidance in CSDS susceptible mice as well as depressogenic behavior in SPT, TST and FST in p11^−/−^ mice	Cheng et al. (2019)
HSV-LS1L-HCN2-eYFP/AAV2-FLEX-HCN2			
AAV2-HCN2-eGFP/HSV-LS1L-HCN2	VTA DA	Reverse CSDS- and CUMS-induced depression- and anxiety-like behaviors in SPT, FST, NSFT and social interaction test	Zhong et al. (2018)
			Friedman et al. (2014)
HCN2 knockdown			
AAV2-HCN2shRNA-eGFP	VTA DA	Decreased sucrose preference in SPT, increased immobility time in FST, increased latency to feed in NSFT and decreased time spent in open arms of EPM	Zhong et al. (2018)
HCN1 knockdown			
LV-HCN1-shRNA	Dorsal hippocampus	Increased the time spent in the centre of OFT and in the open arms of EPM, decreased time spent immobile in FST	Kim et al. (2012)

ChIN, cholinergic interneurons; CUMS, chronic unpredictable mild stress; DA, dopamine; I(h), cationic current through HCN channels; FST, forced swim test; NAc, nucleus accumbens; NSFT, novelty suppressed feeding test; OFT, open field test; SPT, sucrose preference test; VTA, ventral tegmental area.

HCN2 channels’ ability to modulate VTA DA neurons depend on the duration of neuronal excitation: Acute phasic optogenetic stimulation of VTA DA neurons projecting to NAc (20 Hz, 10 min) during a subthreshold social defeat stress increases spontaneous and evoked activity that lasts for 8–12 h after stimulation in parallel with increased depression-like behaviors in social interaction and sucrose preference tests (Chaudhury et al., 2013) (Figure 4). Repetitive optogenetic stimulation of VTA DA neurons for 5 days (20 Hz, 20 min), on the other hand, reduced DA neuron firing rate and displayed antidepressant-like effects in social interaction, forced swim and sucrose preference tests (Friedman et al., 2014) (Figure 4). In line with these observations, both acute injections of HCN channel blockers (ZD7288 or DK-AH 269) into VTA (Cao et al., 2010) and repeated infusions of the I(h) potentiator, lamotrigine into VTA for 5 days showed antidepressant-like effects (Friedman et al., 2014) (Table 2; Figure 4). The group treated with repeated lamotrigine displayed normalization of the hyperactivity of VTA DA neurons (Friedman et al., 2014). A more detailed look at the mechanisms underlying these opposite effects of acute vs repeated manipulations of HCN channels and DA neuron activity showed that I(h) currents were also amplified in social stress resilient mice whose DA neuronal firing rate was comparable to that of controls (Friedman et al., 2014) (Figure 4). Recordings in brain slices of VTA DA neuron demonstrated an increase in K^+^ currents and reduction in firing rate in resilient mice (Friedman et al., 2014). Altogether, these findings suggest that increase in K^+^ currents is a homeostatic plasticity mechanism that stabilizes VTA DA neuronal activity (Figure 4). In support of this proposition, I(h) was found to have a depolarizing effect on the peak voltage of weak EPSPs, whereas it exerted a hyperpolarizing effect on the peak voltage of stronger, but still subthreshold, EPSPs (George et al., 2009). George et al. (2009) showed that blockade of the delayed-rectifier M-type K(+) current resulted in shift of dual I(h) influence on EPSPs to only an excitatory effect, suggesting an interaction between M-type K(+) current and I(h) (George et al., 2009).

Intriguingly, M-type K^+^ (KCNQ) channels have been linked to depression-like behavior and identified as a novel antidepressant drug target (Friedman et al., 2016; Li et al., 2017; Tan et al., 2020; Ren et al., 2021) (Figure 4). RNA sequencing analysis revealed that genes encoding K^+^ channels are differentially expressed in chronically stressed rats (Ren et al., 2021). Transcript levels of KCNQ4 channels that are selectively expressed in VTA DA neurons were negatively correlated with firing rate of VTA DA neurons (Li et al., 2017). Supporting the involvement of KCNQ channels in the pathophysiology of depression, pharmacological activators of KCNQ channels normalized the hyperactivity of VTA DA neurons and alleviated stress-induced depression-like behaviors (Friedman et al., 2016; Li et al., 2017; Ren et al., 2021). A recent open-label clinical study conducted in 18 major depression subjects reported that 10 weeks of treatment with KCNQ channel opener, retigabine, significantly reduced depressive symptoms and decreased functional connectivity between ventral caudate and mid-cingulate and posterior cingulate cortices (Tan et al., 2020).

In contrast to well documented mutations of HCN and KCNQ channel genes in a spectrum of epileptic diseases (Nappi et al., 2020; Rivolta et al., 2020), genetic studies in depressed patients have not conclusively acknowledged a convincing association between single nucleotide polymorphisms (SNPs) in HCN or KCNQ channel genes and depression. Polymorphisms in *HCN4* gene were reported to be associated with a broad spectrum of mood disorders including major depression, bipolar affective disorder and obsessive compulsive disorder (Kelmendi et al., 2011). A study with a larger sample failed to replicate these findings and reported no association between any HCN channel genes studied (HCN1-4) and depression (Mcintosh et al., 2012). Despite the lack of evidence from human genetic studies supporting the involvement of SNPs in HCN or KCNQ channel genes, the above summarized findings in animals suggest that both HCN channels and KCNQ channels are important players of the physiological adaptations to stress, perturbations of which can lead to depression.

### Voltage-Gated Calcium Channels and Two-Pore Domain K+ Channels

Voltage-gated calcium channels and two-pore domain K^+^ channels, the role of which are discussed in the context of migraine in the previous section are also involved in the pathophysiology of mood disorders: Genetic variations in *CACNA1C* gene, which encodes for the alpha subunit of L-type calcium channels (Cav1.2) as well as *CACNA1E*, which encodes for the alpha 1E subunit of R-type calcium channel (Ca_V_2.3 channel) were reported in mood disorders (Ferreira et al., 2008; Psychiatric, 2011; Nurnberger et al., 2014; Howard et al., 2019). Animal studies have confirmed the involvement of *CACNA1C* in the pathophysiolgy of depression: Following CUMS, Cav1.2 levels were found to be increased in the PFC (Bavley et al., 2017). The depression-like behaviors were decreased in both *CACNA1C^+/−^* heterozygous and forebrain specific *CACNA1C* KO mice at baseline and after chronic stress (Dao et al., 2010; Bavley et al., 2017; Kabir et al., 2017; Dedic et al., 2018). In the NAc, on the other hand, CSDS decreased Cav1.2 channel expression in stress susceptible mice and selective KO of *CACNA1C* in the NAc resulted in an increase in susceptibility to subthreshold social defeat stress as well as a reduction in both the time spent sniffing female urine soaked cotton tips and the time spent in open arms of elevated plus maze (Terrillion et al., 2017). In line with these observations, alterations in calcium signaling has long been implicated in the pathophysiology of mood disorders and a recent metaanalysis of 21 studies reported that both basal and stimulated Ca^+2^ levels were found to be higher in platelets and lymphocytes of bipolar affective disorder patients (Harrison et al., 2019). The Ca^+2^ channel blocking properties of mood stabilizer drugs, lithium, valproic acid, carbamazepine and lamotrigine as well as efficacy of verapamil, a Ca^+2^ channel blocker as a mood stabilizing agent support the role of Ca^+2^ in the pathophysiology of mood disorders and point to the promising potential of BBB-permeable Ca^+2^ channel blockers in the treatment of mood disordes (for detailed reviews please see (Cipriani et al., 2016; Dubovsky, 2019; Harrison et al., 2020). Intriguingly, mood stabilizers valproic acid, lamotrigine and carbamazepine also block voltage-gated Na^+^ channels, raising the possibility that their actions might, in part, be attributed to Na^+^ channel blockade (Stahl, 2004). However voltage-gated Na^+^ channels have not been implicated so far in the pathophysiology of depression (Mantegazza et al., 2021).

Among two-pore domain K^+^ channels, the involvement of TWIK-related K^+^ channel-1 (TREK-1) in depression pathophysiology is the most well documented (for detailed reviews see (Borsotto et al., 2015; Djillani et al., 2019)). They are involved in the regulation of plasma membrane potential and excitability similar to HCN channels (Lesage, 2003; Talley et al., 2003). SNPs in *kcnk2*, gene encoding TREK-1 channel, were reported in major depression patients, and some SNPs were found to be associated with antidepressant response and resistance to treatment (Perlis et al., 2008; Liou et al., 2009). TREK-1 inhibitors reversed behavioral despair and decreased sucrose preference induced by CUMS in rats when given intraperitoneously (Ye et al., 2015; Djillani et al., 2017). Deletion of TREK-1 channels resulted in depression-resistant phenotype (Heurteaux et al., 2006). Interestingly selective serotonin uptake inhibitors, fluoxetine, paroxetine and citalopram were reported to block both TREK-1 and TREK-2 mediated currents *in vitro* (Kennard et al., 2005; Kim et al., 2017)*.* Mood stabilizers, lithium, valproic acid and carbamazepine, on the other hand, increased TREK-1 mediated currents without affecting TREK-2 mediated currents (Kim et al., 2017). The therapeutic use of SSRIs in the treatment of both depression and migraine patients may be in part related to their effects on TREK-mediated currents.

In conclusion, mutations detected in familial cases of migraine and polymorphisms creating susceptibility to depression as well as genetic manipulations in animal models of depression have provided novel insight to the pathophysiology of these disorders. Since subtle changes in, for instance, ATP release or K^+^ channel activity may lead to variable consequences within a microcircuit, and hence, in network activity depending on the microcircuit structure and brain region, experimental studies (especially those using isolated preparations) have yielded to varying, sometimes contradictory, results. Therefore, identification of overlapping genetic variants in non-familial forms of migraine and depression can provide further insight to understand the phenotypic traits and role of ion channels, E:I balance, metabolic coupling of astrocytic glycogen to glutamatergic synaptic activity and neuroinflammation in migraine as well as depression, both of which display significant heritability and comorbidity. These findings can also help development of novel therapies for both conditions.
